# GNU polyxmass: a software framework for mass spectrometric simulations of linear (bio-)polymeric analytes

**DOI:** 10.1186/1471-2105-7-226

**Published:** 2006-04-27

**Authors:** Filippo Rusconi

**Affiliations:** 1 CNRS, UMR5153, Paris, F-75231 France; Inserm, U565, Paris, F-75231 France; Muséum national d'Histoire naturelle, Mass spectrometry facility, USM0503, Paris, F-75231, France

## Abstract

**Background:**

Nowadays, a variety of (bio-)polymers can be analyzed by mass spectrometry. The detailed interpretation of the spectra requires a huge number of "hypothesis cycles", comprising the following three actions 1) put forth a structural hypothesis, 2) test it, 3) (in)validate it. This time-consuming and painstaking data scrutiny is alleviated by using specialized software tools. However, all the software tools available to date are polymer chemistry-specific. This imposes a heavy overhead to researchers who do mass spectrometry on a variety of (bio-)polymers, as each polymer type will require a different software tool to perform data simulations and analyses. We developed a software to address the lack of an integrated software framework able to deal with different polymer chemistries.

**Results:**

The GNU polyxmass software framework performs common (bio-)chemical simulations–along with simultaneous mass spectrometric calculations–for any kind of linear bio-polymeric analyte (DNA, RNA, saccharides or proteins). The framework is organized into three modules, all accessible from one single binary program. The modules let the user to 1) define brand new polymer chemistries, 2) perform quick mass calculations using a desktop calculator paradigm, 3) graphically edit polymer sequences and perform (bio-)chemical/mass spectrometric simulations. Any aspect of the mass calculations, polymer chemistry reactions or graphical polymer sequence editing is configurable.

**Conclusion:**

The scientist who uses mass spectrometry to characterize (bio-)polymeric analytes of different chemistries is provided with a single software framework for his data prediction/analysis needs, whatever the polymer chemistry being involved.

## Background

Mass spectrometry has proven essential in structural studies in which biopolymer molecules of a variety of polymer chemistries are involved. Indeed, while proteins were once the main biopolymeric analytes studied by mass spectrometry, oligo(deoxy)ribonucleotides and saccharides also are routinely analyzed today and mass spectrometry is used, for example, for the characterization of DNA-protein complexes or for the gas phase sequencing of saccharides (for reviews, see [1-4]). The current and ever-increasing variety of mass spectrometer designs affords a rather large array of experiments that can be performed on different biopolymers. Thus, the variety of polymer chemistries analyzable by mass spectrometry is compounded by the variety of mass spectrometric experiments, producing an extremely diverse set of mass data to be either predicted or analysed with the help of appropriate software tools. It is noteworthy that, while some experiments are almost completely automatable (like in the case of high-throughput proteomics), a majority of the experiments being performed in mass spectrometry facilities are neither automatable nor high-throughput. For example, one field of biochemistry that has massively benefited from the mass spectrometry improvements over the last ten years is the one involving fine structural characterizations of chemically modified biopolymers, like the post-translational modification studies in protein biochemistry (see [5] for a review). Studies like the ones described in [6] and [7] were not automatable at the time these were performed, and fact is that these are still not automatable today: flexible tools are still needed to help human-driven interpretation of mass spectrometric data obtained on structurally complex biological analytes.

As soon as biopolymers were successfully analyzed by mass spectrometry, a number of tools were made available to perform calculations on proteins or nucleic acids. These tools might be used across the network, such as the tools accessible at the Expasy portal [8] or the ones published in [9]. Locally-installed software is also available, with the massXpert software [10], the GPMAW program (reviewed in [11]) or the SOS program [12]. One problem with the current situation, though, is that each of these programs was designed to work with a given polymer chemistry, thus each time a mass spectrometric analysis is performed on a different biopolymer, a different tool has to be used. The GNU polyxmass software framework was designed to bring a solution to the problem above by providing a number of modules where the user might 1) define any number of polymer chemistries, 2) use polymer chemistry definitions to perform quick mass calculations and 3) graphically edit polymer sequences and perform complex simulations of (bio-)chemical reactions and mass spectrometric experiments.

## Implementation

Creating mass spectrometry software usable with any kind of polymer chemistry did require to elaborate and implement an abstraction layer between on the one hand, the graphical sequence editor and on the other hand, the simulation and mass computation engines. The implementation of this abstraction layer takes the final form of a number of packages, as described below.

### Software development and source tree architecture

The software is developed on a Debian GNU/Linux platform in the "testing/etch" version. This project is structured in a number of packages, two of which are binary packages (*libpolyxmass *and *polyxmass-bin*) and two of which are data packages (*polyxmass-common *and *polyxmass-data*).

#### The binary packages

**Figure 1:**
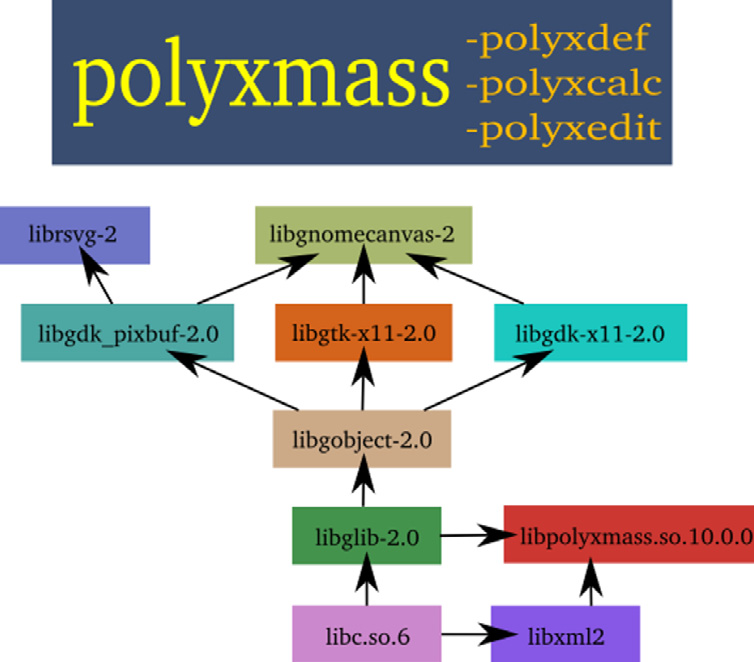
**Software dependencies of the GNU polyxmas project**. The libpolyxmass library has few dependencies while the polyxmass program has more dependencies due to its use of a number of graphical libraries for the window system and the graphical rendering of the polymer sequence.

The *libpolyxmass *and *polyxmass-bin *packages contain code that is exclusively written in the C language. The dependencies of the GNU polyxmass software are described in Figure 1. To maintain a clean separation between low-level code and graphical user interface high-level code, the software project is organized in two distinct code trees: *libpolyxmass *and *polyxmass-bin *(polyxmass binary). Thus, the code in *libpolyxmass *deals with the modelling of all the chemical entities and reactions (atoms, formulas, monomers, oligomers, polymers, cleavages, fragmentations, amongst others) and with a number of housekeeping functions (numerical conversions, parsing of data/configuration files). The code in *polyxmass-bin *is directly related to the graphical user interface functionality, thus relying on a number of graphics-oriented libraries (windowing libraries and libraries for handling scalar vector graphics files and for displaying/handling the polymer sequence).

#### The data packages

The *polyxmass-common *and *polyxmass-data *packages contain polymer chemistry data files and example polymer sequence files (XML format files parsed using the libxml2 library); the former package is considered essential and contains the reference atom definition file (atoms.xml) along with all the files that make together the *"protein" *polymer chemistry definition. The latter package is optional and contains the polymer chemistry definition data for polymer types *"dna"*, *"rna" *and *"saccharide"*.

Configuration data are stored in simple text format files which are read by home-coded functions (in *libpolyxmass*).

Code is subjected to versioning control (using the tla implementation of the GNU arch specification) and is available from 

### Terminological issues

Before describing the details of the software project, it is necessary to put forth terminological decisions we had to take. Indeed, each bio-polymer chemistry has its own set of specific terms to refer to chemical entities in its realm. For example, sugars have "reducing ends" and "monoses", proteins have "N-terminal" and "C-terminal" ends and "residues", and nucleic acids have "3'-OH" and "5'-P" ends and "nucleotides". During the development of GNU polyxmass, we promoted the use of a unified glossary in describing bio-polymer entities, so as to be as generic as possible. "Monomer" refers to the chemical entity that has become part of a polymer sequence chain, that is after its polymerization (equivalent to "residue", in protein chemistry). The denomination of the extremities of the polymer sequence are "left end" and "right end" (equivalent to the protein chemistry "N-terminus" and "C-terminus", respectively). The chemical groups that cap the polymer sequence are called "caps". There is one chemical cap per end. Finally, the equivalent of a protein sequence is called "polymer sequence" and the equivalent of an oligonucleotide is called an "oligomer".

## Results and discussion

The software complexity has been concealed behind an intuitive graphical user interface. GNU polyxmass is mainly operated through a single binary program (polyxmass binary file) that integrates three distinct functionalities organized in modules that can be conveniently called through menu items:

• *polyxdef*: this module lets the user perform the definition of atoms and of brand new polymer chemistries (their file formats are described below);

• *polyxcalc*: this module provides a desktop polymer chemistry-aware mass calculator in which mass computations can be performed without the need to edit polymer sequences;

• *polyxedit*: this module provides the main functionalities of the software framework. It provides a polymer sequence editor in an environment where the user will trigger all the biochemical and mass spectrometric simulations through simple menu interactions.

In the following section, the operation of each module will be reviewed, along with a description of its tasks.

### The polyxdef module

**Figure 2:**
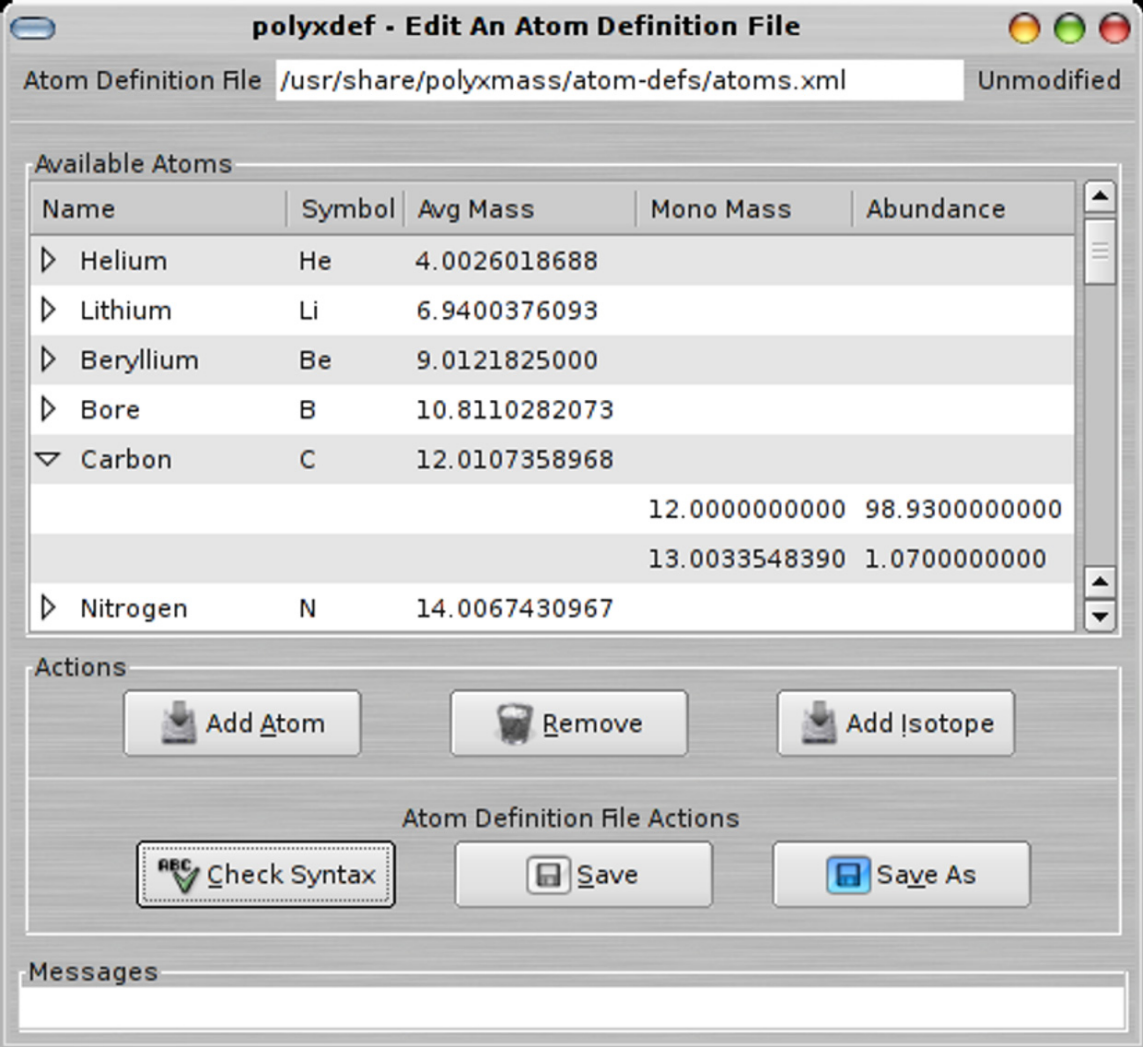
**Atom definitions in the polyxdef module**. The definition of atoms is straightforward: after creation of a new atom "envelope", the user fills-in the isotopic pairs (isotopic mass and relative abundance) required to fully characterize a chemical element. Each time a new isotope is added, the average mass of the chemical element gets recomputed.

**Figure 3:**
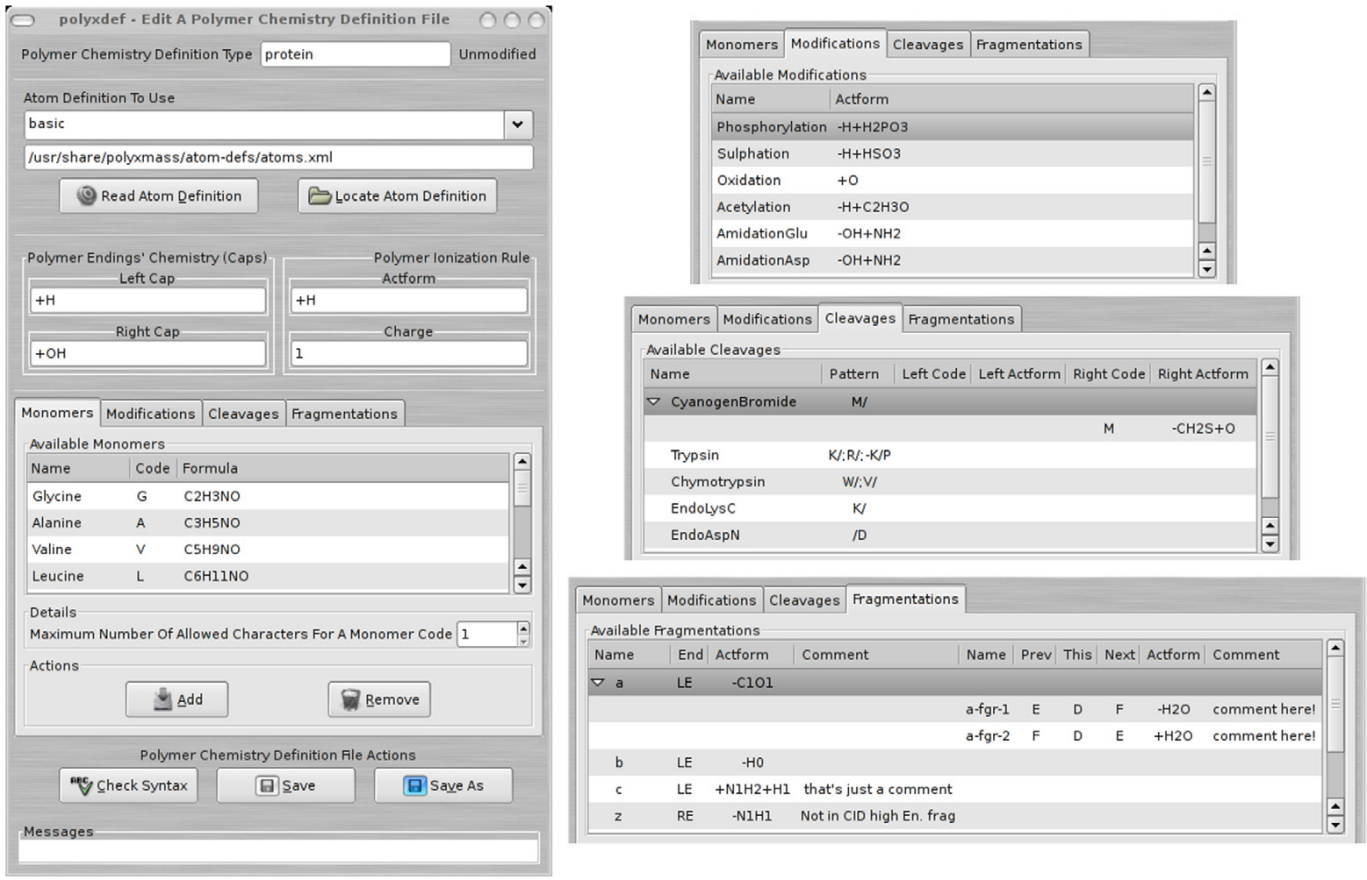
**Polymer chemistry definitions in the polyxdef module**. The elaboration of polymer chemistry definitions is shown in this figure. The definition window is shown on the left, and the screen dumps on the right correspond to the three tabs that are hidden on the left representation of the window. The editing of items in the treeviews is straighforward and takes place in the item rows directly. The example shown pertains to the *"protein" *polymer chemistry definition. See text for details.

The *polyxdef *module is used to perform atom definitions (Figure 2) and polymer chemistry definitions (Figure 3). The use of the module is very intuitive. The formats of the files saved by the *polyxdef *module are described below, along with explanations about the rationale for their specific design.

#### Atom definition file format

No mass is ever hard-coded in the software: in GNU polyxmass, any entity that is ponderable (ie that has a molecular weight) must have a formula associated with it. When masses are computed, formulas are challenged against an atom definition file that lists, for each chemical element, its corresponding isotopic mass/abundance pairs. The following excerpt from the atoms.xml file in the distribution shows the XML structure that was used to allow the dynamic generation of fully characterized atoms.

<atom>

   <name>Hydrogen</name>

   <symbol>H</symbol>

   <isotope>

         <mass>1.0078250370</mass>

         <abund>99.9885000000</abund>

   </isotope>

   <isotope>

         <mass>2.0141017870</mass>

         <abund>0.0115000000</abund>

   </isotope>

</atom>

Each chemical element has a monoisotopic mass that corresponds to the lightest isotope's mass; the average mass is computed by considering all the isotopes of the chemical element. The atom average masses are computed dynamically once, when the atom definition file is loaded from disk. There can be any number of distinct atom definition files on the system.

#### Polymer chemistry definition file format

The polymer chemistry definition constitutes the most evident mechanism by which to achieve a full uncoupling between the sequence editor and the mass calculation engine. Each polymer chemistry type (*"protein"*, for example) has such a definition file, where all aspects of its chemistry are documented. The following code excerpts were taken from the protein.xml file in the distribution. They illustrate the way in which the polymer chemistry definition is parted into XML nodes documenting the different chemical entities that make such chemical definition: monomers, modifications, chemical/enzymatic cleavages, gas-phase fragmentations, chemical end caps, ionization rule. All of these entities are reviewed below.

##### Monomers

<codelen>3</codelen>

<monomers>

   <mnm>

      <name>Glycine</name>

      <code>G</code>

      <formula>C2H3NO</formula>

   </mnm>

   ...

</monomers>

Because polymers are the result of the concatenation of monomers, listing the monomers that might enter in the composition of the polymer sequence is obviously the first step in defining a polymer chemistry. Monomers are defined as having a name, a code and a formula. The <codelen> element specifies the number of characters allowed to define a monomer code. This is a value that has a polymer chemistry definition scope. One common arbitrary limitation with mass spectrometry programs is that the editing of the sequence can be performed using only one letter-long monomer codes (thus limiting the number of codes to 26). GNU polyxmass removes this limitation with the design and implementation of sophisticated algorithms that make it possible to use any number of alphabetical characters to form a monomer code. The syntactic rule governing the formation of a code is that the first letter must be uppercase and all the remaining ones lowercase. This is particularly useful when working with modified monomers, in which case using more letters may help differentiating monomers with different modifications. For example, with a <codelen> element of value 2, 'Y' could be used for un-modified tyrosinyl residues, "Yp" after its phosphorylation and "Ys" after its sulphation.

##### Modifications

<modifs>

   <mdf>

      <name>Phosphorylation</name>

      <actform>-H+H2PO3</actform>

   </mdf>

   ...

</modifs>

Modifications also are polymer chemistry-specific. Note the peculiar formula which characterizes them, which is called an "actform", short for "action-formula". The net mass change, upon modification of the polymer sequence, is applied after computation of the net formula by subtracting the atoms prefixed with the '*-*' sign from the atoms prefixed with the '+' (if at all) sign. This notation has the advantage of being more meaningful from a chemical reaction standpoint, as it closely represents it, with the interplay of the leaving and the entering groups at the target biopolymer molecule level.

##### Cleavage specifications

<cleavespecs>

   <cls>

      <name>CyanogenBromide</name>

      <pattern>M/</pattern>

      <clr>

         <re-mnm-code>M</re-mnm-code>

         <re-actform>-CH2S+O</re-actform>

      </clr>

   </cls>

   <cls>

      <name>Trypsin</name>

      <pattern>K/;R/;-K/P</pattern>

   </cls>

   ...

</cleavespecs>

Enzymatic/chemical cleavage agents (<cls> elements) belong to the polymer chemistry definition. The example of cyanogen bromide, as a cleaving chemical agent, illustrates the flexibility of the definition language: when a protein sequence is cleaved with cyanogen bromide, the methionyl residue that got cleaved at its C-terminal side is chemically transformed to a homoseryl residue. That reaction is described using the actform "*-*CH_2_S + O" and is only applied to the generated peptides if the methionyl residue is located at its C-terminus (that is, the right end of the oligomer; see the <re-mnm-code> element above).

##### Fragmentation specifications

<fragspecs>

   <fgs>

      <name>a</name>

      <end>LE</end>

      <actform>-C101</actform>

      <fgr>

         <name>a-fgr-1</name>

         <actform>-H2O</actform>

         <prev-mnm-code>E</prev-mnm-code>

         <this-mnm-code>D</this-mnm-code>

         <next-mnm-code>F</next-mnm-code>

         <comment>comment here!</comment>

      </fgr>

      ...

   </fgs>

   ...

   <fgs>

      <name>imm</name>

      <end>NE</end>

      <actform>-C1O1+H1</actform>

   </fgs>

</fragspecs>

Gas-phase fragmentation patterns ("specifications"; <fgs> elements) are defined using a powerful syntax. A fragmentation specification lists the following data:

• name: this is the name of the fragmentation pattern, and will be used to craft each fragment's name;

• end: this datum specifies which side of the polymer chain will make the fragment after the fragmentation occurred. In protein chemistry, the 'a' fragmentation pattern describes the ions corresponding to the sequence portion left of the fragmentation location [13]. In GNU polyxmass, three values are acceptable: "LE" for left end, "RE" for right end and "NE" for specific fragmentation cases like immonium ions in protein chemistry;

• fgr: any number of fragmentation rules ("fragrule"; <fgr> elements) can be defined that will be applied to the fragment ions if sequence topological conditions are verified. The example a-fgr-1 fragrule states that if fragmentation occurs at a monomer of code 'D' (<this-mnm-code> element) *AND *that the previous monomer in sequence has a code 'E' *AND *that the next monomer in sequence has a code 'F', then the actform "-H2O" should be applied to the fragment ion;

This fragmentation specification grammar is flexible enough to allow the description of highly complex fragmentation patterns such as those observed in saccharidic gas-phase fragmentations [14]. Indeed, fragmentation patterns that depend on the identity of the monomer at which the fragmentation occurs (and, sometimes, also the identity of the bordering monomers) can be modelled using the provided grammar. For example, the "a-B" fragmentation pattern observed in oligonucleotide fragmentation is easily supported in GNU polyxmass. Internal fragments generation is not supported yet.

##### End cap chemistry

<leftcap>+H</leftcap>

<rightcap>+OH</rightcap>

The mere concatenation of monomers only yields a residual chain of monomers, and not a polymer in its finished polymerization state. Thus, it is necessary to provide chemical definitions of how the residual monomeric chain gets capped to finish the polymerization. In protein chemistry, the left (<leftcap> element) and right (<rightcap> element) caps are the proton and the hydroxyl group, respectively.

##### Ionization rule

<ionizerule>

   <actform>+H</actform>

   <charge>1</charge>

   <level>1</level>

</ionizerule>

A default ionization rule is defined in the <ionizerule> element according to the following scheme:

• actform: this is the chemical representation of the ionization reaction. For proteins, protonation is by far the most often used ionization mechanism, while for a number of polymers (including synthetic ones) the best ionization mechanism is not protonation, but cationization with metal ions;

• charge: this is the charge that is brought to the polymer sequence after the ionization reaction took place. Protonation-based ionizations bring a charge of one;

• level: this is the number of times that the ionization reaction should be performed on the polymer sequence. Usually mono-protonation is the preferred ionization level for peptides.

Once a polymer chemistry has been defined, its definition file might be used to compute masses with the calculator (*polyxcalc *module) or to edit sequences and to trigger biochemical and mass spectrometric simulations on these (*polyxedit *module).

### The polyxcalc module

**Figure 4:**
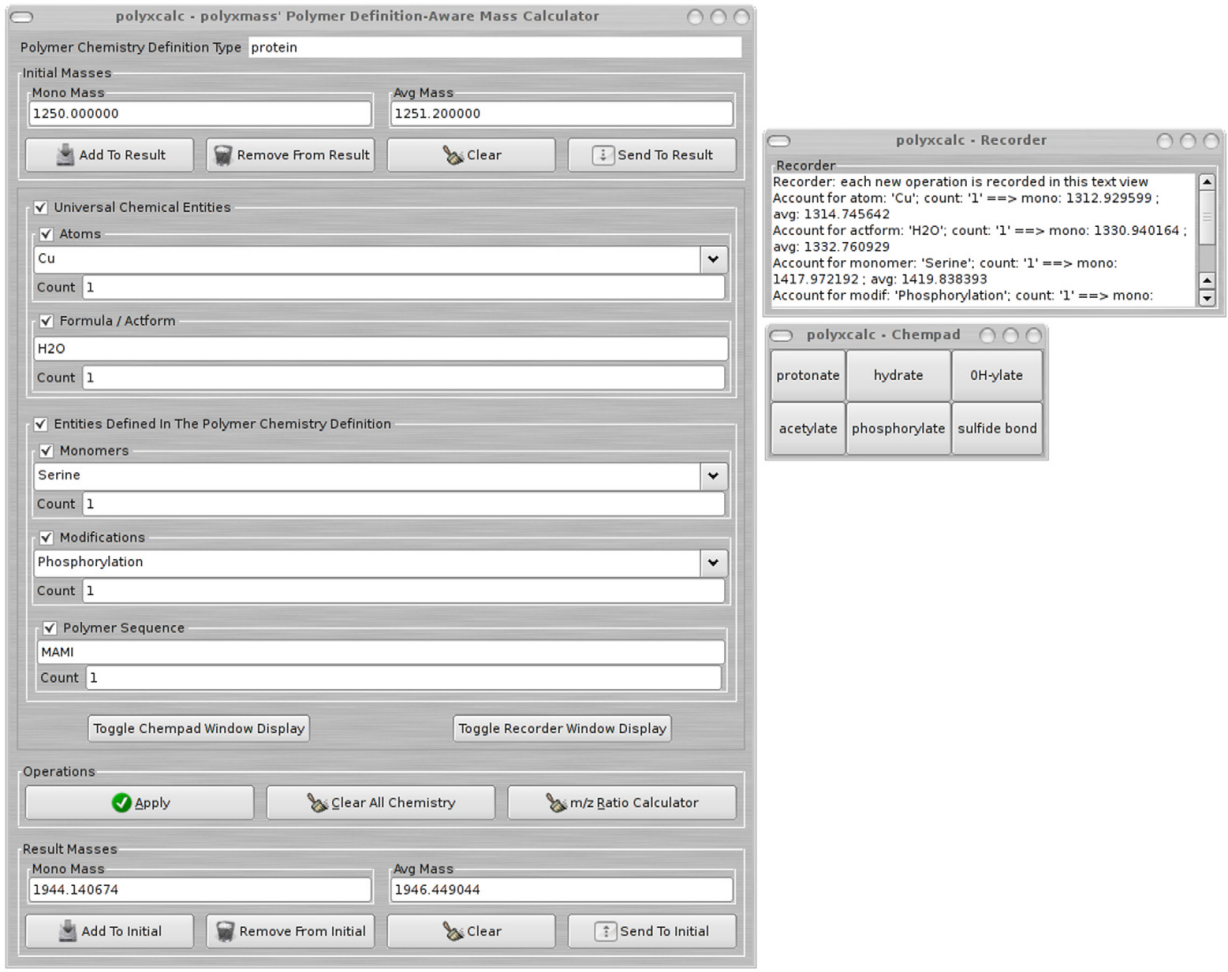
**The polyxcalc programmable mass calculator**. The mass calculator is polymer chemistry definition-aware, and in this figure it is represented pre-loaded with the *"protein" *polymer chemistry definition (top of the left window). When, upon opening of the calculator, a polymer chemistry definition is loaded, its chemical entities (monomers and chemical modifications, as shown) become available in the calculator's widgets; otherwise only atoms and formulas can be used. The calculator is programmable, and a simple syntax allows the definition of the layout of a "chemical pad" to perform quick chemical calculation tasks in the calculator.

The *polyxcalc *module is shown in Figure 4. By default, any computation might be performed by using atoms and formulas. However, if a polymer chemistry definition is loaded into the calculator, then all the chemical entities defined in it are made available for the calculations. This is illustrated in the figure, where the *"protein" *polymer definition was loaded. It is possible to feed the calculator with initial masses (upper part of the window) so that they are taken into account during the calculations. Interestingly, it is possible to program the calculator with a simple text file, which leads to the creation of a "chemical pad" (chempad; right side of the figure). Clicking onto one chempad button triggers the computation that was programmed for it. There is no limitation on the number of buttons available in the chempad. There might be one chempad configuration file per polymer chemistry definition or a single default configuration file that will be used whatever the polymer chemistry being loaded in the calculator. A scrolling logbook recorder ensures that all the operations are logged in their smallest detail.

### The polyxedit module

The *polyxedit *module is the most featureful module of the program. This section first describes the critical mechanisms that ensure that all the simulations performed with polymer sequences are using the proper polymer chemistry definition. Next, the different functionalities presented by *polyxedit *will be reviewed briefly.

#### Chemical consistency between a sequence and the biochemical and mass spectrometric simulations

**Figure 5:**
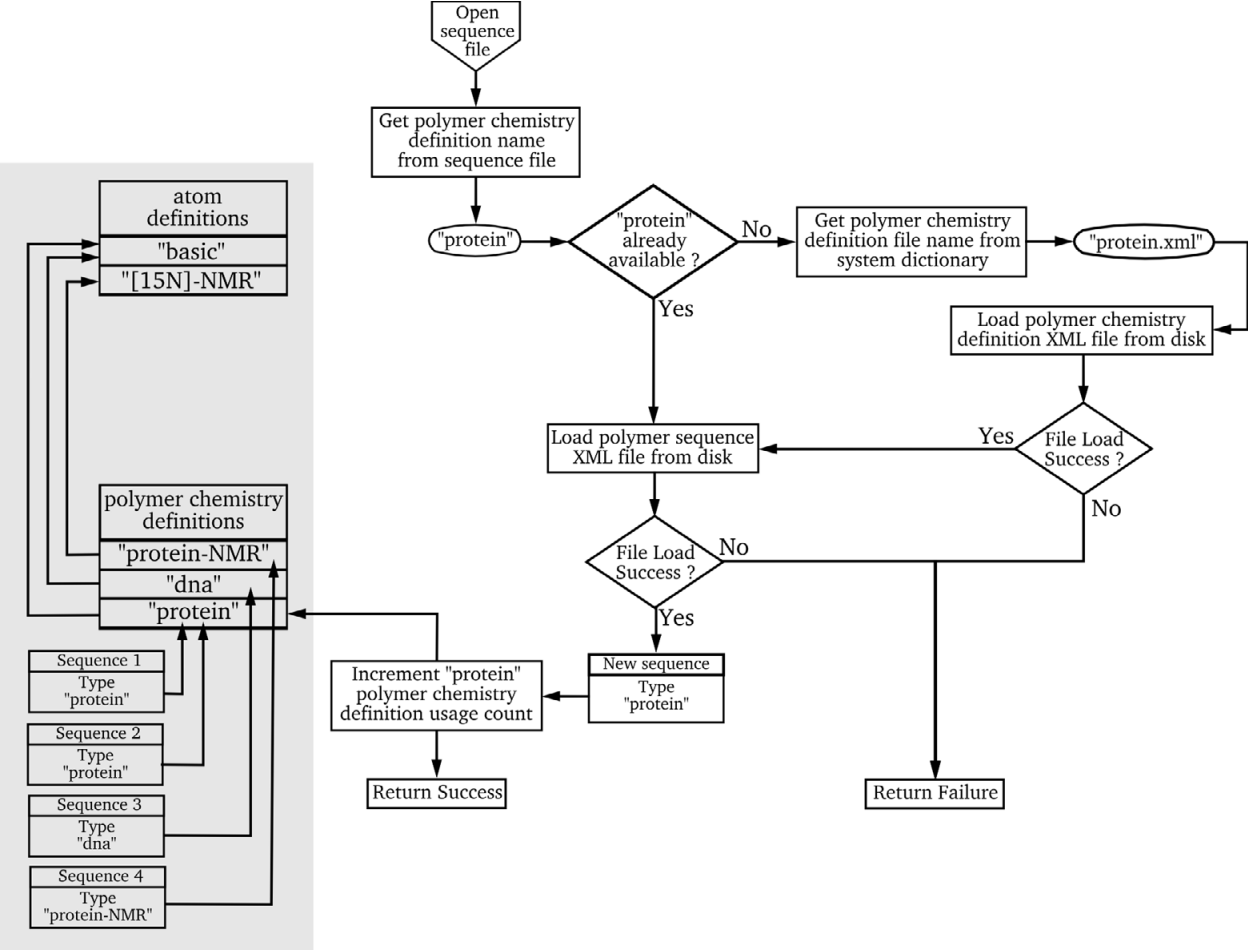
**Each polymer sequence has a connection with a polymer chemistry definiton**. It is crucial, for the proper functioning of the software, that each polymer sequence be permanently connected to a valid polymer chemistry definition. This is required so that the biochemical/mass spectrometric simulations be performed using proper chemical entities. This figure illustrates the algorithm which is used in the software to make sure that any polymer sequence being loaded in the sequence editor has a corresponding polymer chemistry definition associated to it and that this definition is actually available on the system.

Because GNU polyxmass allows any number of polymer sequences of any polymer chemistry to be open simultaneously, it is essential that the thread connecting a given polymer sequence to its related polymer chemistry definition be permanently in order. This essential trait of GNU polyxmass is illustrated in Figure 5 where the left shaded area represents the memory status of the program, with four polymer sequences opened (Sequence 1 to Sequence 4). The first two sequences are of the same *"protein" *polymer chemistry definition, while the other ones are of polymer chemistry definitions *"dna" *and *"protein-NMR"*. Each sequence stores a pointer to its corresponding polymer chemistry definition (arrows). All the polymer chemistry definitions in use at any given point in time are stored in a global array (denoted "polymer chemistry definitions" in the figure). Reference counting management of the polymer chemistry definitions ensure the tightest memory use. Each polymer chemistry definition is required to have an atom definition with which to calculate masses and therefore must store a pointer to its corresponding atom definition (arrows). Because any number of polymer sequences of any polymer chemistry definition might be open at any given point in time, it is necessary to store all the available atom definitions in an array *ad hoc *(denoted "atom definitions", top of the shaded area of the figure).

The non-shaded part of the figure shows the mechanisms by which the program ensures, upon opening of a polymer sequence file, that the proper polymer chemistry definition is available, either already in memory or accessible on the disk. Indeed, a polymer chemistry definition is absolutely required in order to interpret correctly the sequence data contained in the sequence file: a given sequence–like "ATGC", for example–might mean different things depending on the polymer chemistry for which it was edited (that is, this sequence could be a nucleic acids sequence or a protein sequence).

The action "Open sequence file" triggers code that will inspect the polymer sequence file in search for a <type> XML element that documents the polymer chemistry definition of the sequence. That discovery process yields, in our example, *"protein" *as the polymer chemistry definition type. Thus, the sequence being opened is a protein sequence. The algorithm checks if the polymer chemistry definition is already available in the global array of polymer chemistry definitions. If so, there is no need to load that chemistry definition file into memory and the program goes on to load the polymer sequence. If not, the program has to first load the polymer chemistry definition from a file. The name of that file is discovered by reading a dictionary file that makes the link between a polymer chemistry definition (*"protein"*) and the location of its corresponding file on disk. When the polymer chemistry definition file is successfully loaded, it is made available to the whole program by storing its pointer in the global array of polymer chemistry definitions mentioned above. The loading of the polymer sequence can continue, and if successful, the sequence will point to the polymer chemistry definition just loaded. This mechanism enforces the rule that any given sequence loaded from disk has to be attached to the proper polymer chemistry definition, thus ensuring that the biochemical and mass spectrometric simulations that are performed on the sequence actually make use of the proper chemical entities as defined in the right polymer chemistry definition file.

#### Graphical editing of polymer sequences

**Figure 6:**
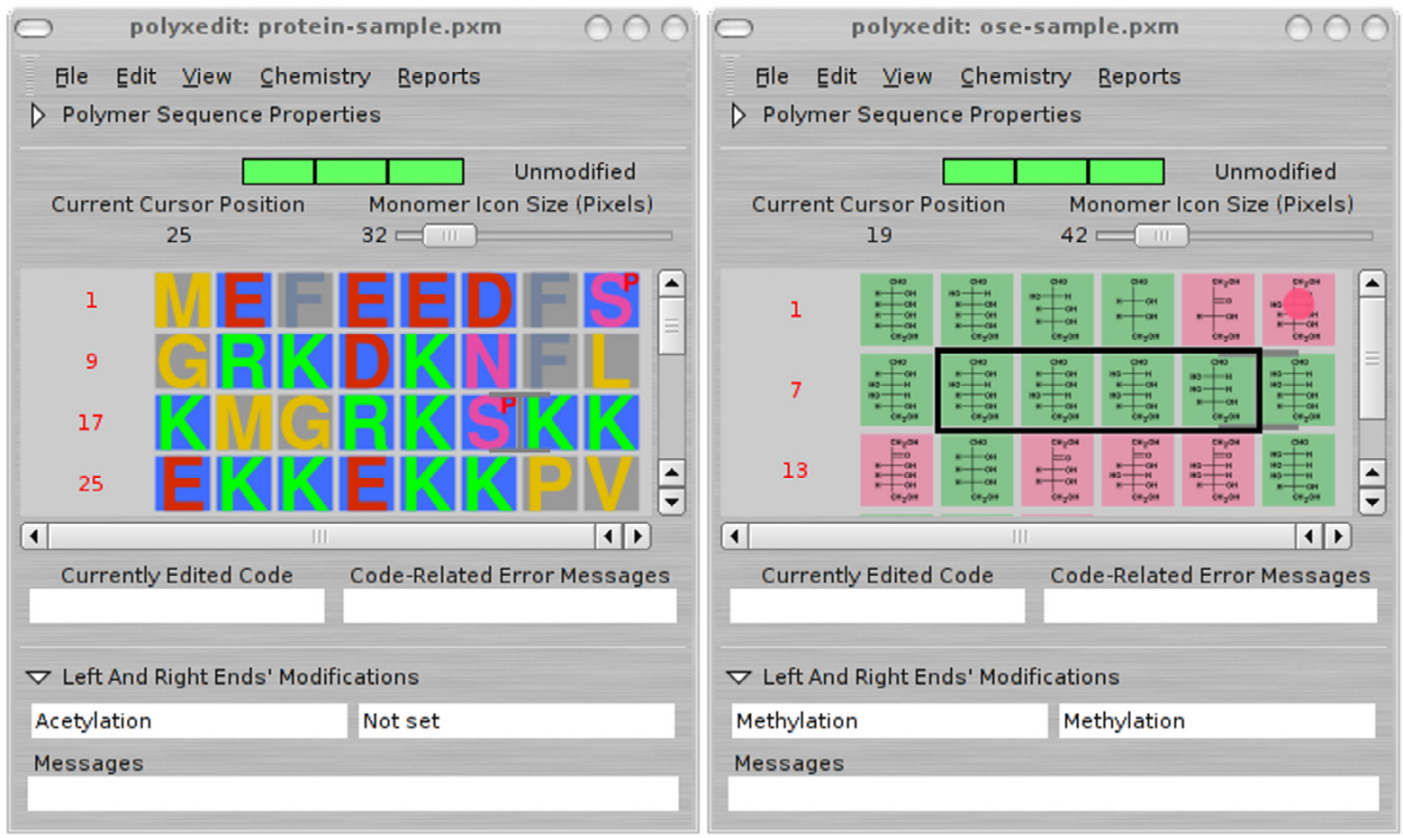
**The polyxedit module: the sequence editor**. This figure illustrates one of the original features of the polymer sequence editor. The user might configure the software in such a way that the graphical representation of the monomers of a polymer sequence be performed in distinct manners depending on the polymer chemistry. As visible on the figure, the left window represent a protein sequence with each monomer being represented using its 1-letter code. The right window represent an oligosaccharide, for which each monomer is represented with its own chemical formula. The configurability of the display is almost unlimited. Operating the editor feels exactly like using an ordinary text editor. Selection of sequence portions leads to the drawing of a selection polygon with a transparent area (shown in the right sequence editor window screen dump).

Polymer sequence editing will certainly be reminiscent of any simple text file editing task; however, the internals of the polymer sequence editor are rather innovative. Indeed, from a graphical perspective, the polymer sequence editor enables a complete decoupling between the polymer sequence as it is stored in the polymer sequence XML file and the way it gets represented graphically. This is shown in Figure 6, which shows two polymer sequences: a *"protein" *sequence on the left and a *"saccharide" *sequence on the right. The mechanism that allows this differential display of sequences of different polymer chemistry definitions is based on dictionary files. Each polymer chemistry definition has a dictionary (monicons.dic) that lists, for each monomer code, the corresponding graphics file to be used to render it graphically in the sequence editor (the monomer pixmaps are called "monomer icons"). The user is empowered to define any aspect of that graphical rendering since the graphical files can be freely modified or created anew. Noteworthy, if the graphics files are stored in the scalar vector graphics (SVG) format, then the program will be able to scale the sequence representation still retaining the best definition for the screen it is displayed onto (see the right sequence in Figure 6, which has monomer icons 42 pixels wide, while the left sequence has monomer icons 32 pixels wide). This effect is obtained by rendering in memory–with the new resolution–each SVG file as a portable network graphics (PNG) pixmap. The PNG pixmaps are then graphically composited onto the sequence editor canvas to reconstitute the polymer sequence.

Chemical modifications of the monomers can be rendered graphically by compositing a transparent pixmap onto the monomer icon to be modified. For example, in the left sequence of Figure 6, the 'S' monomer icon was modified by compositing onto it a transparent pixmap with a red 'P' graphical element (the seryl residue is phosphorylated). This is unlimitedly configurable, because the transparent graphics files representing chemical modifications are–like described above– freely modifiable or can be created anew.

#### Available simulations

**Figure 7:**
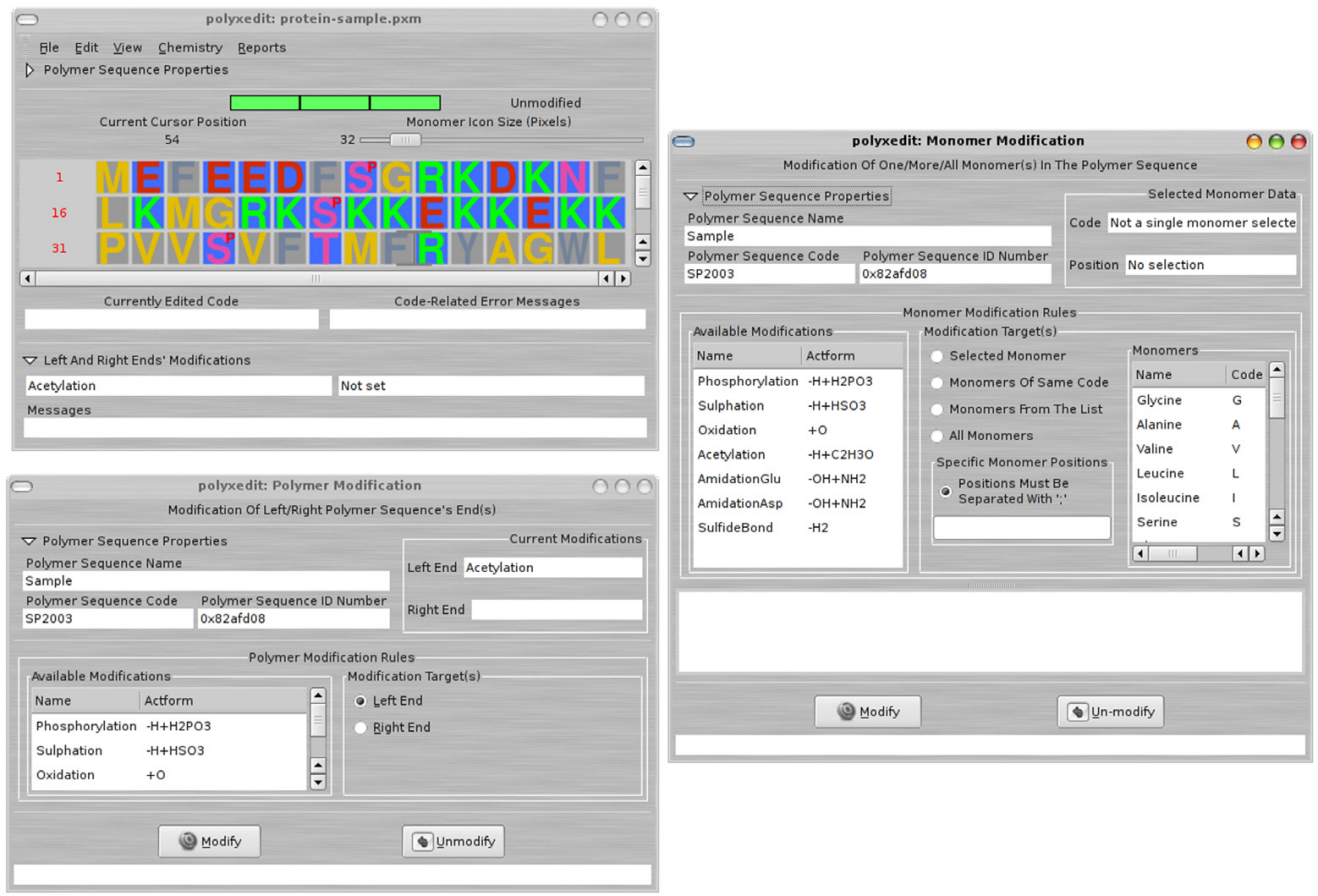
**Chemical modifications of the polymer or of monomers**. There are two sorts of modifications: 1) the modifications that involve the polymer sequence as a whole (these happen at the left/right ends of the polymer sequence; see left bottom window), 2) the modifications that are set to monomers according to highly flexible criteria, described in the right window, in the "Modification Target(s)" section. The latter modifications are intrinsic to the monomer(s) to which they are set: if the monomer is erased from the sequence, the modification is destroyed also.

**Figure 8:**
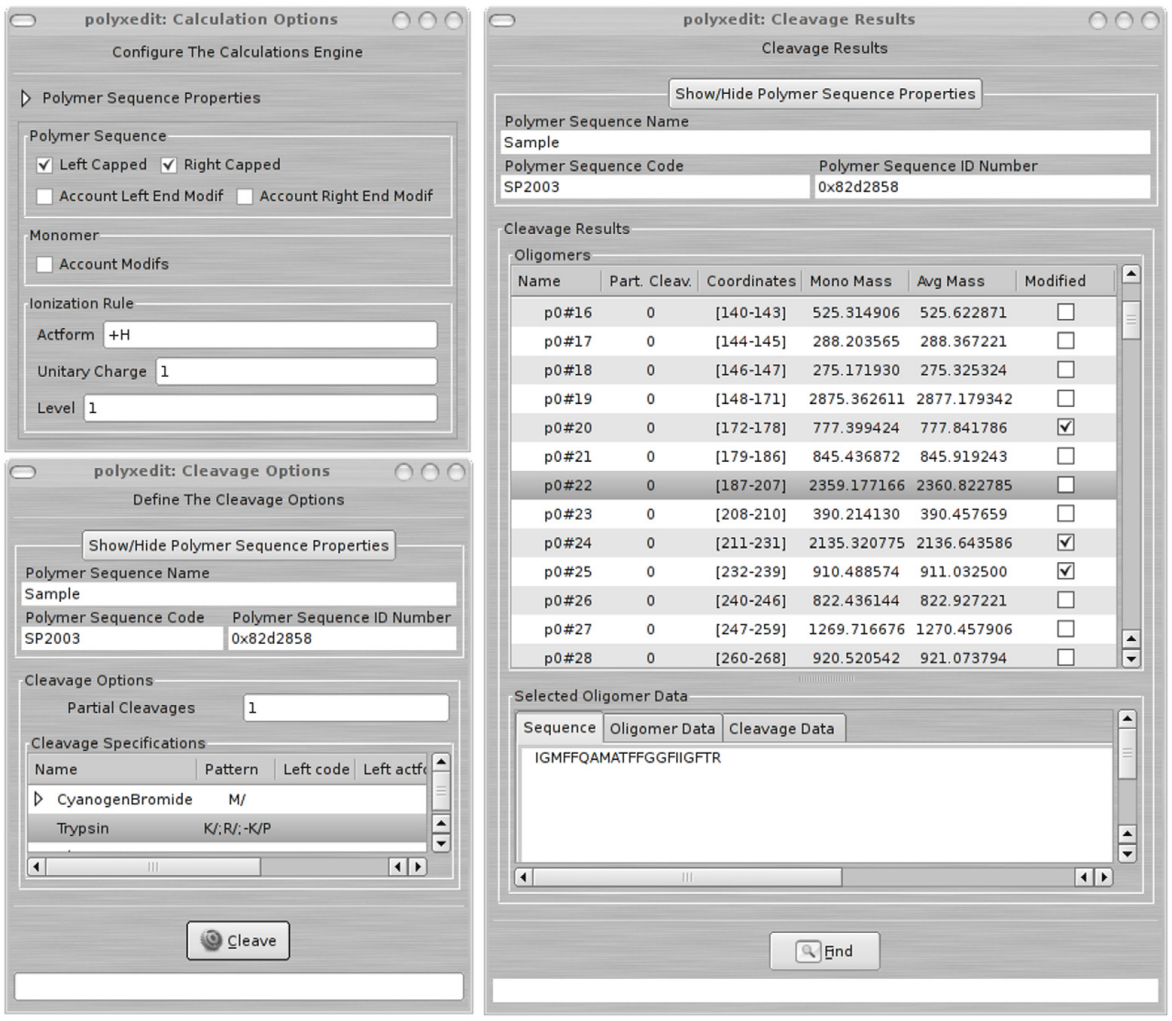
**Cleavage of a polymer sequence : options and results**. This figure illustrates the process involved in cleaving a polymer sequence (a protein) with an enzyme (trypsin). The options for the cleavage are set in the lower left window. Upon cleavage, all the peptides will be generated (those resulting from full sequence cleavage plus the set of peptides corresponding to a single partial cleavage, as required in the options window). The masses of the oligomers (peptides) generated will be computed according to the configuration of the mass calculation engine shown on the upper left window screen capture. The cleavage results are displayed on the right window. When a peptide is clicked (one treeview row), its data are displayed below the list of peptides; if the item is double-clicked, its corresponding sequence is highlighted in the polymer sequence editor window. It is notheworthy that searching for specific masses in the newly generated set of oligomers is allowed by clicking onto the "Find" button.

**Figure 9:**
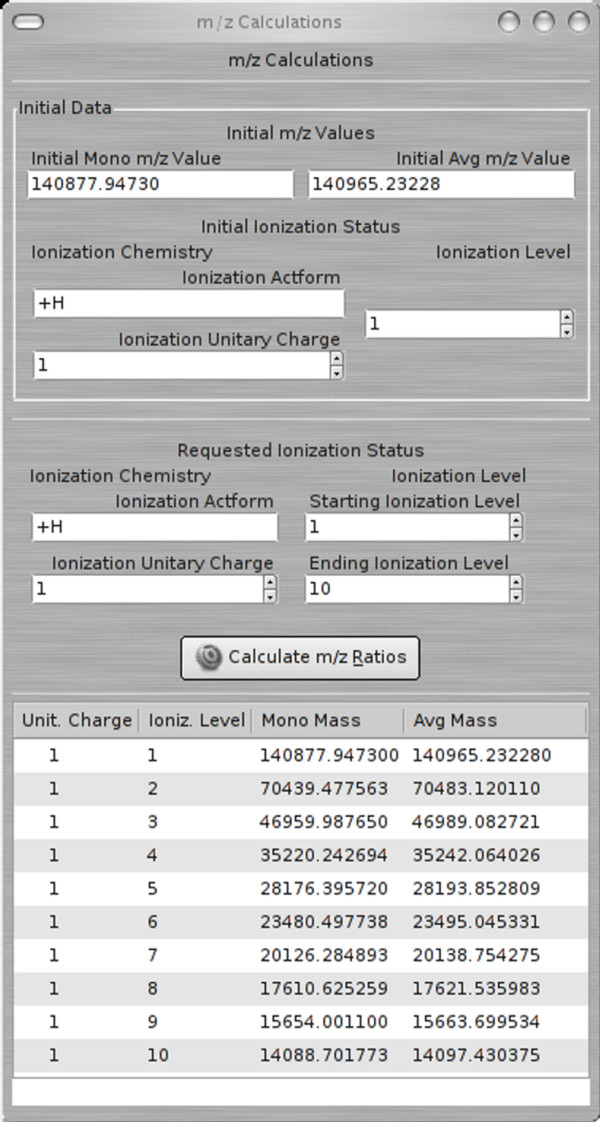
**Mass-to-charge (m/z) ratio calculations**. This figure shows how to perform m/z calculations starting from an analyte with a given m/z value for a given ionization agent (a proton, in our example). It is possible to ask for m/z ratios to be computed for any z value interval. Further, if the ionization has changed (by switching from protonation to cationization, for example), it is possible to stipulate the new ionization agent and its mass is taken into account for the calculation of the m/z values in the asked z range. The results are shown in the bottom part of the window.

**Figure 10:**
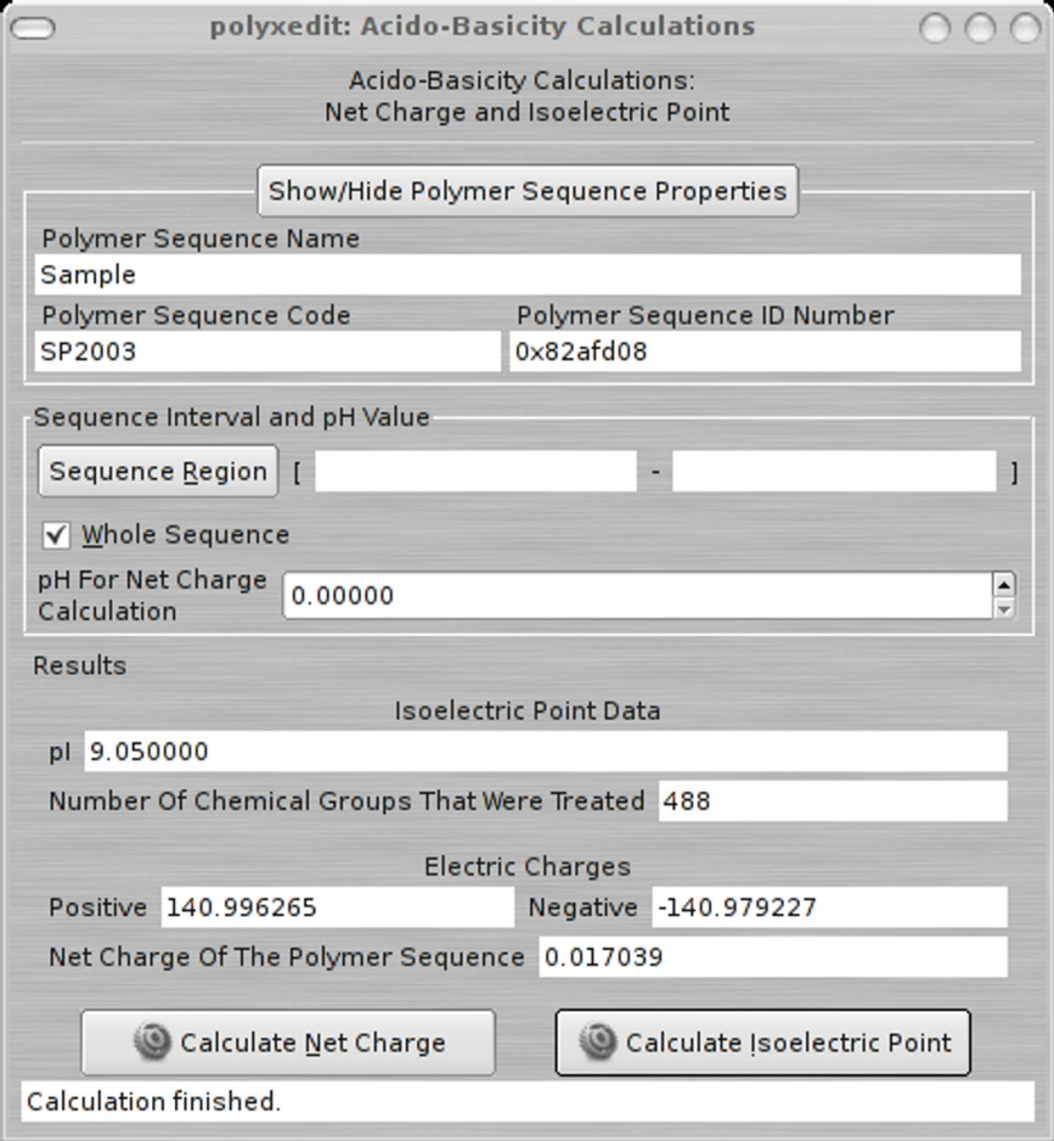
**Isoelectric point and net charge calculations**. This figure shows that two calculations are available: the calculation of the net charge of a polymer sequence when it is at a given pH value (left button) or the calculation of the isoelectric point of the polymer sequence (right button; our example). It is possible to make calculations on the whole polymer sequence or–by selecting a portion of the sequence in the editor–to restrict the calculation to that specific sequence region.

When a polymer sequence is opened in the sequence editor, as shown in Figure 6, all the simulations and computations are automatically made available in the editor menus. The simulations and computations that are available through the sequence editor menus are:

• Chemical modifications of individual monomers or of the polymer sequence as a whole on its left/right ends (see Figure 7 for an overview);

• Chemical/enzymatic cleavage of a polymer sequence, with automatic fully configurable mass calculations (see Figure 8);

• Gas-phase fragmentation of an oligomer, with automatic fully configurable mass calculations. The process here is in any aspect comparable to what was described above for the cleavage of polymer sequences;

• Mass-to-charge (m/z) ratio calculations with the ability to change the chemical ionization agent on the fly (see Figure 9);

• Calculation of the composition of a polymer sequence (both monomeric and elemental);

• Isoelectric point calculations of polymer sequences, optionally taking into account chemical modifications (see Figure 10 for an overview);

• Matching operations between data extracted from a real mass spectrum and data generated *in silico *by GNU polyxmass. The procedure is to deisotope the mass spectrum and to compute the centroid for the remaining peaks. All the centroid peaks are listed in a peak list. Such list is then fed into GNU polyxmass and all the masses it contains can be matched with a GNU polyxmass-generated theoretical mass spectrum.

• Annotation of the whole polymer sequence or of single monomers;

• Fully customizable find/replace operations;

• Full reporting of all the results in a spreadsheet-friendly ASCII format.

## Conclusion

The GNU polyxmass software is an integrated mass spectrometric software framework that allows biochemists and massists to perform biochemical and mass spectrometric simulations on polymer sequences of any polymer chemistry. The software design that drove the implementation of GNU polyxmass constitutes an improvement over the current situation, in which each time a mass spectrometric experiment is performed on a different polymer chemistry (protein, nucleic acid or saccharide, for example), the data it yields have to be analyzed using a different software tool. We find GNU polyxmass useful in our day-to-day mass spectrometric work on proteins and peptides, serving as a mass data prediction and mass data analysis tool. We also have used GNU polyxmass successfully as an education tool, as it behaves exactly like a mass spectrometer, allowing a great number of mass spectrometry concepts to be experimented virtually. This software framework was implemented with configurability and flexibility in mind and with the idea that no arbitrary limit should be imposed on the user. It is published under a Free Software license in the hope to form a community around it, to continue and further its adoption and development as a research and education tool.

## Future developments

The GNU polyxmass software framework will be made extensible by use of the Python scripting language. Further, it is envisaged to integrate a mass spectrum visualization tool that will be able to render mass spectrometric data files of recently published XML-based open formats mzXML [15] and mzData [16,17]. Easy graphical differential display between theoretically and experimentally obtained mass spectra might thus be made possible in GNU polyxmass. Features like cross-links and ramification of polymers are being elaborated upon and might find their way in future releases.

## Availability and requirements

• Project name: GNU polyxmass;

• Project home page: 

• Operating system(s): GNU/Linux, UNIX, Mac OS X/Fink;

• Programming language: C;

• License: GNU General Public License;

## Authors' contributions

The author developed the ideas presented in this paper, did the software project analysis, designed the data file formats, programmed all the software, did the testing, and wrote the report.
